# Supplementary material for: Comparative analysis of the liver transcriptome in the red-eared slider Trachemys scripta elegans under chronic salinity stress
Source: PeerJ. 2019 Mar 21;7:e6538. doi: 10.7717/peerj.6538 (PMC6431541; doi:10.7717/peerj.6538)
Supplement: Table S1 [file peerj-07-6538-s001.docx]

| **Table S1 Major GO terms related to osmotic regulation in 0 vs 5 psu groups** | | | |
| --- | --- | --- | --- |
| **Description** | ***P*_bonferroni** | **Type** | **Differential genes** |
| Ion binding | 3.88E-05 | molecular_function | COX1;c101564_g1;ADH4;TRIM39;CYP2P;c105226_g1;c105433_g1;c106588_g1;SNAI2,SLUG;c107478_g1;DSC1;TAT;ADCK,ABC1;TNK1;c111614_g1;ALB;K06911;c112636_g1;ACTB_G1;DDR2,TKT;KRAB;RASD2;RASD2;CDHE,CDH1;CDH3;c115683_g7;TRIM29,ATDC;c117980_g4;c119888_g1;c120156_g1;STK32,YANK;CYP2C;ADH1_7;SIK;ALPK;E1.2.1.5;CRYAA;GPX4;c156641_g1;c168787_g1;APOE;CYTB,petB;glnA,GLUL;c19896_g1;SOCS1,JAB;CRYL1;EPHA8,EEK;TEX14,SGK307;MYL7;ACADVL;c72315_g1;TUBA;COX2;E2.7.3.2;c86748_g1;KRAB;c88272_g1;c88272_g2;c90496_g1;PKD1L2;STK33;HOMER;ASPN;c99149_g1;c99623_g2 |
| Ion transmembrane transport | 0.000195 | biological_process | COX1;TRPA1,ANKTM1;COX3;ATPeF0A,MTATP6,ATP6;CHRNA4;KCNH5;SLC26A9;COX2 |
| Hydrogen ion transmembrane transporter activity | 0.00029 | molecular_function | COX1;COX3;ATPeF0A, MTATP6,ATP6;COX2 |
| Hydrogen ion transmembrane transport | 0.000332 | biological_process | COX1;COX3;ATPeF0A,MTATP6,ATP6;COX2 |
| Hormone activity | 0.00106 | molecular_function | ADM;c893_g1;NPPA;ACDC |
| Inorganic ion transmembrane transport | 0.00201 | biological_process | COX1;COX3;ATPeF0A,MTATP6,ATP6;KCNH5;SLC26A9;COX2 |
| Monovalent inorganic cation transport | 0.00596 | biological_process | COX1;COX3;ATPeF0A,MTATP6,ATP6;KCNH5;SCN1B;COX2 |
| Anion binding | 0.00643 | molecular_function | ADH4;c105433_g1;TAT;ADCK,ABC1;TNK1;c111614_g1;ALB;c112636_g1;ACTB_G1;DDR2,TKT;RASD2;RASD2;c120156_g1;STK32,YANK;SIK;ALPK;E1.2.1.5;GPX4;c156641_g1;APOE;glnA,GLUL;c19896_g1;CRYL1;EPHA8,EEK;TEX14,SGK307;ACADVL;c72315_g1;TUBA;E2.7.3.2;c88272_g1;c88272_g2;c90496_g1;STK33;HOMER;c99623_g2 |
| Cellular amino acid metabolic process | 0.00659 | biological_process | GST,gst;TAT;MTHFD2;c113444_g1;c120156_g1;STK32,YANK;SIK;ALPK;E1.2.1.5;GPX4;PTS-HPR;glnA,GLUL;OAZ3;E2.3.1.7;CRYL1;E2.7.3.2;STK33;HOMER;K14165 |
| Ion transport | 0.00735 | biological_process | COX1;TRPA1,ANKTM1;SLC6A5S;COX3;c1641_g1;ATPeF0A,MTATP6,ATP6;CHRNA4;KCNH5;SLC26A9;APOE;SCN1B;COX2 |
| Ion transmembrane transporter activity | 0.00816 | molecular_function | COX1;TRPA1,ANKTM1;SLC6A5S;COX3;ATPeF0A, ATP6;CHRNA4;KCNH5;SLC26A9;COX2 |
| Nitrogen compound metabolic process | 0.0168 | biological_process | c106312_g1;c106644_g2;GST,gst;c107959_g1;TAT;MTHFD2;FBP,fbp;c112105_g3;c112323_g1;c113444_g1;RASD2;RASD2;c115683_g7;E3.1.27.1;c120156_g1;STK32,YANK;SIK;K16330;ALPK;E1.2.1.5;c124849_g1;GPX4;c132576_g1;c1641_g1;PTS-HPR;ATPeF0A,MTATP6,ATP6;glnA,GLUL;OAZ3;c207168_g1;E2.3.1.7;CRYL1;TUBA;E2.7.3.2;STK33;HOMER;K14165;c99023_g1;c99149_g1 |
| Inorganic cation transmembrane transport | 0.0338 | biological_process | COX1;COX3;ATPeF0A,MTATP6,ATP6;KCNH5;COX2 |
| Inorganic cation transmembrane transporter activity | 0.0354 | molecular_function | COX1;SLC6A5S;COX3;ATPeF0A,MTATP6,ATP6;KCNH5;COX2 |
| Cation transmembrane transport | 0.0378 | biological_process | COX1;COX3;ATPeF0A,MTATP6,ATP6;KCNH5;COX2 |
