# Supplementary material for: Comparative analysis of the liver transcriptome in the red-eared slider Trachemys scripta elegans under chronic salinity stress
Source: PeerJ. 2019 Mar 21;7:e6538. doi: 10.7717/peerj.6538 (PMC6431541; doi:10.7717/peerj.6538)
Supplement: Table S2 [file peerj-07-6538-s002.docx]

| **Table S2. Major GO terms related to osmotic regulation in 0 vs 15 psu groups** | | | |
| --- | --- | --- | --- |
| **Description** | ***P*_bonferroni** | **Type** | **Differential genes** |
| Ion binding | 2.96E-07 | molecular_function | c100305_g1;COX1;c101564_g1;MFI2;TRIM39;CYP2K;EPHA7,EHK3,HEK11;CYP2P;KLF15;c105226_g1;c105433_g1;MDK;KRAB;c106494_g1;SNAI2,SLUG;c107478_g1;DSC1;TAT;c109536_g1;htpG,HSP90A;MYO7;TNK1;PLOD2;c111564_g1;ALB;EPHB2,ERK,DRT;K06911;MYL3;RNF170;ACTB_G1;c114593_g4;RASD2;c115288_g1;TRIB;EPHA1,EPH;LHCB1;THBS1;TRIM29,ATDC;AGXT2L1,ETNPPL;AGXT2L1,ETNPPL;c116330_g1;c116515_g1;c116999_g1;adk,AK;NR2A1,HNF4A;c117849_g1;c118484_g1;c118989_g1;c119497_g1;INSRR;ZC3H12,MCPIP;GCK;STK32,YANK;SLC25A23S;PLA2G,SPLA2;SIK;CYP17A;ARL4;c123809_g3;NPNT;c124238_g1;CRYAA;COX1;c155439_g1;c156641_g1;c168787_g1;CYTB,petB;glnA,GLUL;c19896_g1;SOCS1,JAB;CRYL1;TNNC1;TEX14,SGK307;MYL7;ACADVL;E2.7.3.2;CETN2;c86748_g1;FN1;c88272_g1;c88272_g2;c88310_g1;c90496_g1;c93858_g1;argG, ASS1;c99623_g2 |
| Anion binding | 3.90E-05 | molecular_function | c100305_g1;EPHA7,EHK3,HEK11;c105433_g1;MDK;c106494_g1;TAT;htpG, HSP90A;MYO7;TNK1;PLOD2;ALB;EPHB2,ERK,DRT;ACTB_G1;c114593_g4;RASD2;TRIB;EPHA1,EPH;THBS1;AGXT2L1,ETNPPL;AGXT2L1,ETNPPL;c116999_g1;adk,AK;c117849_g1;INSRR;GCK;STK32,YANK;SIK;ARL4;ARL4;c123809_g3;c156641_g1;glnA,GLUL;c19896_g1;CRYL1;TEX14,SGK307;ACADVL;E2.7.3.2;FN1;c88272_g1;c88272_g2;c88310_g1;c90496_g1;argG,ASS1;c99623_g2 |
| Iron ion binding | 0.000159 | molecular_function | COX1;MFI2;CYP2K;CYP2P;c107478_g1;PLOD2;CYP17A;c124238_g1 |
| Calcium ion binding | 0.000395 | molecular_function | c101564_g1;c105226_g1;DSC1;c109536_g1;MYL3;THBS1;c116330_g1;c118484_g1;c119497_g1;SLC25A23S;PLA2G,SPLA2;NPNT;c155439_g1;TNNC1;MYL7;CETN2;c86748_g1;c93858_g1 |
| Proton transport | 0.00128 | biological_process | COX1;COX3;c131564_g1;ATPeF0A,MTATP6,ATP6;ND2 |
| Hydrogen transport | 0.00135 | biological_process | COX1;COX3;c131564_g1;ATPeF0A,MTATP6,ATP6;ND2 |
| Transmembrane transport | 0.00202 | biological_process | COX1;SLC7A9,BAT1;SLC6A5S;CD79B,IGB;SLC38A2,SNAT2;MIA40,CHCHD4;TRPA1,ANKTM1;c118570_g1;SLC5A9,SGLT4;SLC25A23S;COX3;ATPeF0A, MTATP6, ATP6;c75960_g1 |
| Hormone activity | 0.00224 | molecular_function | CD79B, IGB;NPPA;RLN;ACDC |
| Monovalent inorganic cation transport | 0.00335 | biological_process | COX1;CD79B,IGB;COX3;c131564_g1;ATPeF0A,MTATP6,ATP6;ND2;SCN1B |
| Ion transport | 0.0046 | biological_process | COX1;MFI2;SLC7A9,BAT1;SLC6A5S;CD79B,IGB;SLC38A2,SNAT2;TRPA1,ANKTM1;c118570_g1;COX3;c131564_g1;c1641_g1;ATPeF0A,MTATP6,ATP6;ND2;SCN1B;c75960_g1 |
| Metal ion binding | 0.0112 | molecular_function | COX1;c101564_g1;MFI2;TRIM39;CYP2K;CYP2P;KLF15;c105226_g1;KRAB;SNAI2,SLUG;c107478_g1;DSC1;c109536_g1;PLOD2;c111564_g1;K06911;MYL3;RNF170;c115288_g1;LHCB1;THBS1;TRIM29,ATDC;c116330_g1;c116515_g1;NR2A1,HNF4A;c118484_g1;c118989_g1;c119497_g1;ZC3H12,MCPIP;SLC25A23S;PLA2G,SPLA2;CYP17A;NPNT;c124238_g1;CRYAA;c155439_g1;c168787_g1;CYTB,petB;SOCS1,JAB;TNNC1;MYL7;CETN2;c86748_g1;c93858_g1 |
| Hydrogen ion transmembrane transporter activity | 0.012 | molecular_function | COX1;COX3;ATPeF0A,MTATP6,ATP6 |
| Cation binding | 0.0122 | molecular_function | COX1;c101564_g1;MFI2;TRIM39;CYP2K;CYP2P;KLF15;c105226_g1;KRAB;SNAI2,SLUG;c107478_g1;DSC1;c109536_g1;PLOD2;c111564_g1;K06911;MYL3;RNF170;c115288_g1;LHCB1;THBS1;TRIM29,ATDC;c116330_g1;c116515_g1;NR2A1,HNF4A;c118484_g1;c118989_g1;c119497_g1;ZC3H12,MCPIP;SLC25A23S;PLA2G,SPLA2;CYP17A;NPNT;c124238_g1;CRYAA;c155439_g1;c168787_g1;CYTB,petB;SOCS1,JAB;TNNC1;MYL7;CETN2;c86748_g1;c93858_g1 |
| Hydrogen ion transmembrane transport | 0.0135 | biological_process | COX1;COX3ATPeF0A,MTATP6,ATP6 |
| Ion transmembrane transport | 0.0369 | biological_process | COX1;SLC7A9,BAT1;CD79B,IGB;SLC38A2,SNAT2;TRPA1,ANKTM1;COX3;COX1;ATPeF0A, MTATP6, ATP6 |
| Transaminase activity | 0.0406 | molecular_function | TAT;AGXT2L1, ETNPPL;GPT, ALT |
